# Antibacterial and anti-biofilm activity of mouthrinses containing cetylpyridinium chloride and sodium fluoride

**DOI:** 10.1186/s12866-015-0501-x

**Published:** 2015-08-21

**Authors:** Joe Latimer, Jodie L Munday, Kara M Buzza, Sarah Forbes, Prem K Sreenivasan, Andrew J McBain

**Affiliations:** Manchester Pharmacy School, The University of Manchester, Oxford Road, Manchester, M13 9PT UK; Colgate-Palmolive Technology Center, Piscataway, NJ USA

## Abstract

**Background:**

Cetylpyridinium chloride (CPC) and sodium fluoride augment oral hygiene by inactivating bacteria and inhibiting enamel demineralisation, respectively. However, there are few reports in the literature documenting the antibacterial efficacy of their combined use in mouthrinses. We have used six experimental systems to compare the antibacterial effects of mouthrinses containing 0.075 % CPC (test rinse, TR) or 0.075 % CPC with sodium fluoride (test fluoride rinse, TFR).

**Results:**

Effects against planktonic bacteria were determined using viable counting (for *Streptococcus mutans* and salivary bacteria), a redox dye (for *Actinomyces viscosus* and salivary bacteria) and viable counting (for *ex vivo* oral rinses). Effects against saliva-derived biofilms were quantified using confocal microscopy and differential viable counting. Inhibition of biofilm formation was evaluated by pre-treating hydroxyapatite coupons with mouthrinses prior to inoculation. Otherwise-identical controls without CPC (control rinse and control fluoride rinse, CR and CFR, respectively), were included throughout. Compared to the controls, TFR and TR demonstrated significant antimicrobial effects in the redox assays, by viable counts (>3 log reductions) and in oral rinse samples (>1.25 log reductions, *p* < 0.05). TFR and TR also significantly reduced the viability of oral biofilms. Pre-treatment of hydroxyapatite with TFR and TR significantly inhibited biofilm formation (>3 log difference, *p* < 0.05). Overall, there were no consistent differences in the activities of TR and TFR.

**Conclusions:**

Sodium fluoride did not influence the antibacterial and anti-biofilm potency of CPC-containing formulations, supporting the combined use of CPC and sodium fluoride in mouthrinses to control oral bacteria and protect tooth enamel.

## Background

Dental caries is a major public health problem throughout the world [1, 2]. Extensive research has indicated that the major cause of caries is the acidification of tooth surfaces following fermentation of dietary sugars by cariogenic bacteria which accumulate on the surfaces of teeth within dental plaque [3]. If the pH of the tooth surface drops below a critical value, thought to be approximately 5.5, enamel will begin to demineralise [4] eventually resulting in the formation of carious lesions. Commonly-implicated causative bacteria include *Streptococcus mutans* and homofermentative lactobacilli (as reviewed by Marsh [5]). *Actinomyces* species are also believed to be significant contributors, particularly to root caries [6–8]. Thorough mechanical removal of plaque twice daily with a fluoride-containing toothpaste is a commonly-taught method of caries prevention. However, studies show that incidence of dental caries remains high [9, 10], suggesting that such regimens are commonly not strictly adhered to.

Mouthrinses containing antibacterial compounds may augment routine oral hygiene measures by inactivating bacteria remaining in the mouth after brushing [11–13] and by inhibiting their regrowth and reattachment to tooth surfaces. A variety of antibacterial formulations have been produced, incorporating actives such as chlorhexidine, ethanol and essential oils, with differing levels of reported antibacterial effects and oral substantivity [14–18]. Cetylpyridinium chloride (CPC), a cationic quaternary ammonium compound, has been demonstrated in clinical and *in vitro* studies to inactivate oral bacteria, reducing plaque and gingivitis [19–25].

It is believed that the combined use of fluoride with an antibacterial compound is beneficial in the prevention of caries [26] due to simultaneous strengthening of enamel and inactivation of bacteria. Antibacterial agents inactivate oral bacteria, decreasing the bacterial burden, thereby reducing net acid production and moderating the pH drop following sugar consumption (as reviewed by Gilbert *et al.* [27]). Fluoride acts primarily by decreasing the pH at which enamel demineralizes. Tooth enamel is composed of crystals of hydroxyapatite, a mineral form of calcium apatite (Ca_10_(PO_4_)_6_(OH)_2_). Free fluoride ions can adsorb to hydroxyapatite crystals, inhibiting demineralisation during acid challenge and enhancing remineralisation when pH levels subsequently rise [28, 29]. Fluoride may also reduce acid production by inhibiting bacterial glucose metabolism and thus reducing acidogenesis and the associated enrichment of aciduric species in plaque [30]. There have been recent concerns that the use of mouthrinses immediately following brushing may remove residual fluoride [31]. Incorporation of fluoride into mouthrinses may compensate for this loss by effectively delivering fluoride which is then retained in saliva post-rinsing [32, 33].

Clinical research has demonstrated that experimental mouthrinse formulations containing both sodium fluoride and CPC are effective in reducing the accumulation of supragingival plaque bacteria [24] and are as effective as a fluoride-only rinse in inhibiting enamel demineralisation *in vitro* [34]. This suggests that CPC does not interfere with the effects of fluoride. However, no comprehensive studies are available in the literature that have investigated the antibacterial efficacy of CPC-based mouthrinses with or without added fluoride. Further, the activities of formulations and active ingredients are commonly assessed using a single methodological approach or selected species of interest [35–38]. The current investigation therefore used multiple approaches to compare the antibacterial efficacy of mouthrinses formulated with 0.075 % CPC, alone or in combination with 225 ppm sodium fluoride. Activities against planktonic bacteria were assessed using pure cultures of cariogenic species, oral bacterial consortia and *ex-situ* human oral samples. Activities against established plaques and on the inhibition of plaque formation were also assessed.

## Results

### Test formulations rapidly inactivate established cultures of oral bacteria

**Fig. 1:**
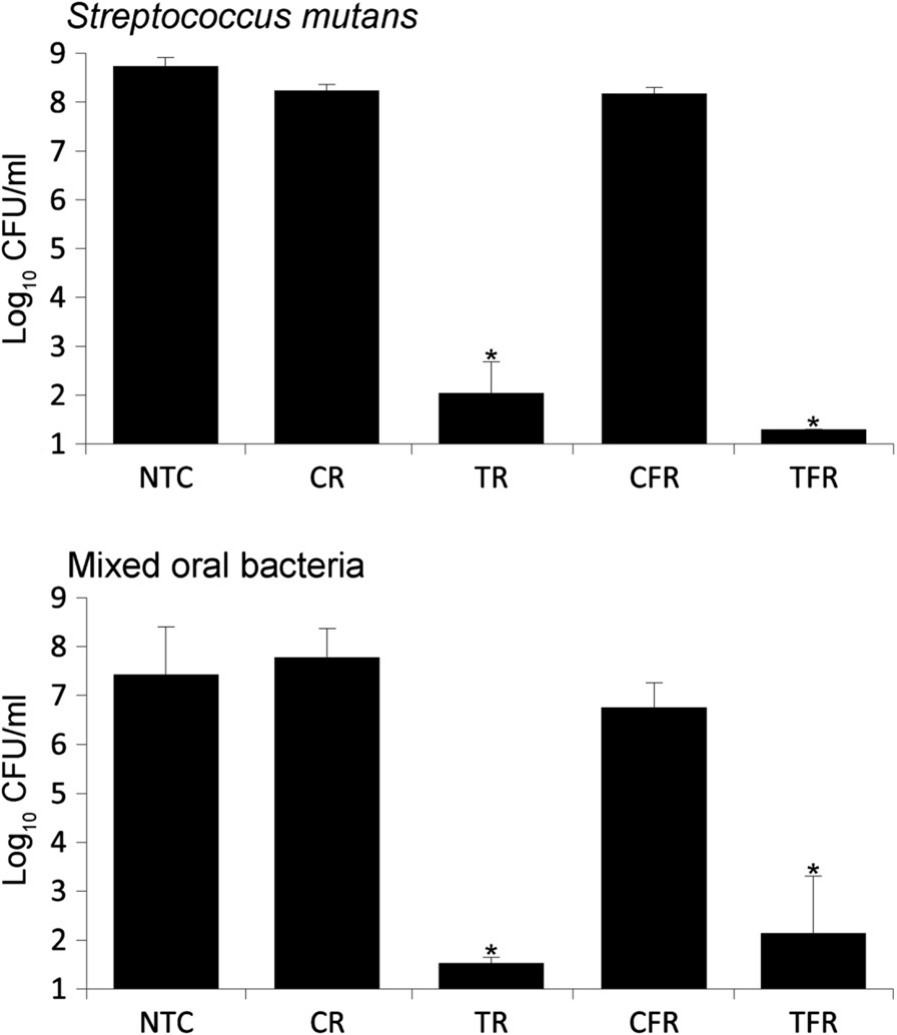
Viability of established cultures, as determined using viable counts, following exposure to test rinses. Pelleted cultures of *Streptococcus mutans* (*upper panel*) and mixed salivary bacteria (*lower panel*) were exposed to mouthrinse formulations (final concentration, 100 %) containing CPC (TR) or CPC and fluoride (TFR) in triplicate. Control rinses without CPC (CR and CFR, respectively) and a treatment-free control (NTC) were also included. Data show bacterial viability, as determined by viable counts. Both test rinses caused significant reductions in viability compared to their respective controls in three separate experiments (*, *p* < 0.05, *n* = 3).

Bactericidal effects were assessed by viable counts following exposure to test rinses. These experiments revealed that TR and TFR caused 6.2 Log_10_ and 6.9 Log_10_ reductions in viable counts of *Streptococcus mutans*, respectively, compared to their respective controls (Fig. 1). A modest but significant increase in bactericidal efficacy was observed in the formulation containing fluoride in comparison with the non-fluoridated rinse. Significant effects of both formulations were also observed against mixed salivary bacteria (6.2 Log_10_ and 4.6 Log_10_ reductions for TR and TFR respectively), but with significantly increased efficacy observed in the non-fluoridated formulation.

**Fig. 2:**
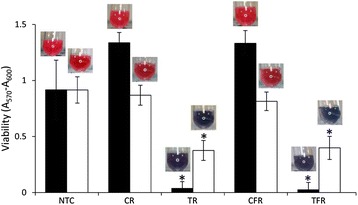
Viability of established cultures, as determined using a redox dye, following exposure to test rinses. Cultures of *Actinomyces viscosus* (*black bars*) and mixed salivary bacteria (*white bars*) were exposed to mouthrinse formulations (final concentration, 10 % v/v) containing CPC (TR) or CPC and fluoride (TFR) in triplicate. Control rinses without CPC (CR and CFR, respectively) and a treatment-free control (NTC) were also included. Data show mean viability (background-corrected A_570_-A_600_), as determined with a redox indicator dye. Representative images are included. Both test rinses caused significant reductions in viability compared to their respective controls in three separate experiments (*, *p* < 0.05, *n* = 3)

The short-term effects of test rinses on the redox activity of established cultures of oral bacteria were assessed using a resazurin-based redox dye. Both of the test rinses caused significant reductions in the viability of *A. viscosus* and mixed salivary bacteria within 10 min (Fig. 2.). This effect was more marked in cultures of *A. viscosus* than salivary bacteria. There were no significant differences between the effects of TR and TFR. There was no significant difference in bacterial viability following treatment with the control rinses.

### Test formulations inactivate bacteria from *ex vivo* oral rinse samples

The effects of test rinses on the viability of *ex situ* human oral bacteria were assessed by exposing oral rinse samples from healthy volunteers, *ex vivo*, to test formulations (Table 1). TR and TFR both caused greater than a1.0 Log reduction in comparison to their respective controls. There was no significant difference between the control rinses and the untreated control. There was no significant difference in the viability of samples treated with the fluoridated and non-fluoridated rinses.

**Table 1:** Viability of human oral rinse samples following *ex-vivo* exposure to test rinses

Treatment	Mean counts (Log_10_ cfu/ml; SDs are given in parenthesis)	Log_10_ difference from control	*P* value (vs control)
NTC	7.92 (0.25)	0.16 (vs CR)	0.34
		−0.094 (vs CFR)	0.64
CR	8.08 (0.25)	na	na
TR	6.72 (0.63)	1.37 (vs CR)	0.02
CFR	7.64 (0.50)	na	na
TFR	6.36 (1.1)	1.27 (vs CFR)	0.04

Oral rinse samples from five healthy volunteers were exposed to mouthrinse formulations (final concentration, 5 % v/v) containing CPC (TR) or CPC and fluoride (TFR). Control rinses without CPC (CR and CFR, respectively) and a treatment-free control (NTC) were also included. Mean viable count data are shown. Both test rinses caused significant reductions in viability compared to their respective controls in samples from five volunteers (*, *p* < 0.05, *n* = 5). na, not applicable

### Test formulations inactivate *in vitro* oral biofilms

**Fig. 3:**
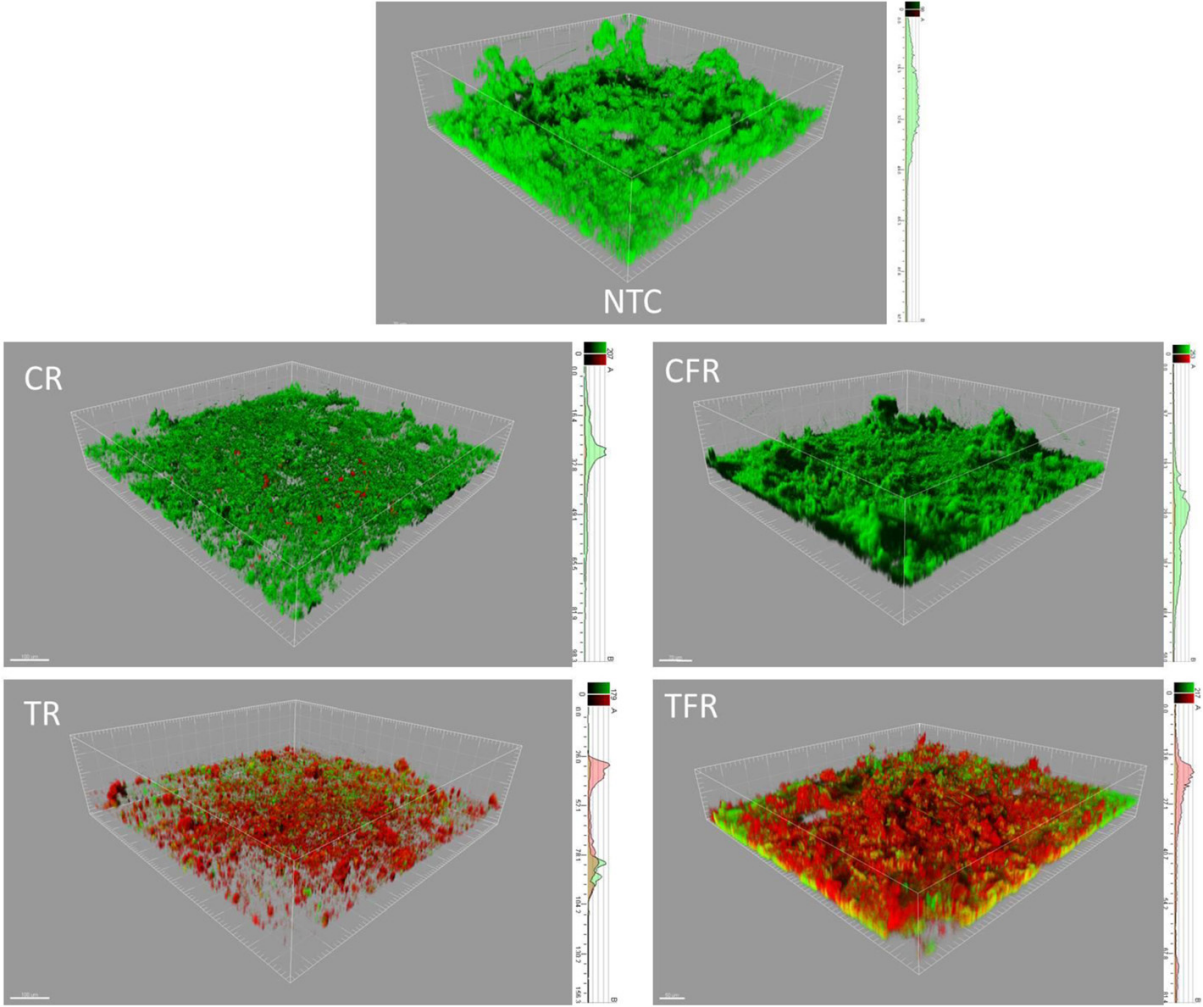
Confocal microscopy of oral biofilms following exposure to test rinses. Biofilms were cultivated on hydroxyapatite surfaces inoculated with saliva and exposed to mouthrinse formulations (final concentration, 100 %) containing CPC (TR) or CPC and fluoride (TFR). Control rinses without CPC (CR and CFR, respectively) and a treatment-free control (NTC) were also included. Biofilms were then treated with a fluorescent viability stain and visualised by confocal microscopy. Three-dimensional projections show representative images of plaques and two-dimensional plots show representative depth profiles plotted through the deepest section of biofilm. Red, non-viable mass; green, viable mass. Higher proportions of red fluorescence among biofilms treated with test rinses indicate widespread inactivation of bacteria through their depths. Each grid represents a 776.5 μm^2^ area of biofilm, each large grid square representing 51.77 μm^2^. Depth profiles measure 97.7, 98.3, 58.0, 156.3 and 81.4 μm for NTC, CR, CFR, TR and TFR, respectively

Plaques were cultivated under anaerobic conditions to reflect the anoxic environments of these complex communities. Plaques were visualised by confocal microscopy following single exposures of test rinses, using a viability stain containing a combination of SYTO9 and propidium iodide to differentiate viable and non-viable cells. Images and resulting depth profiles revealed that test rinses caused marked reductions in the viability of oral biofilms; even at depth, as indicated by a distinct increase in the relative levels of red fluorescence through their depths (Fig. 3). The appearance and profiles of the biofilms exposed to the control rinses resembled much more closely those of the treatment-free control, exhibiting mainly green fluorescence with small areas of non-viable mass which were localised predominantly in the centres of larger clusters.

**Fig. 4:**
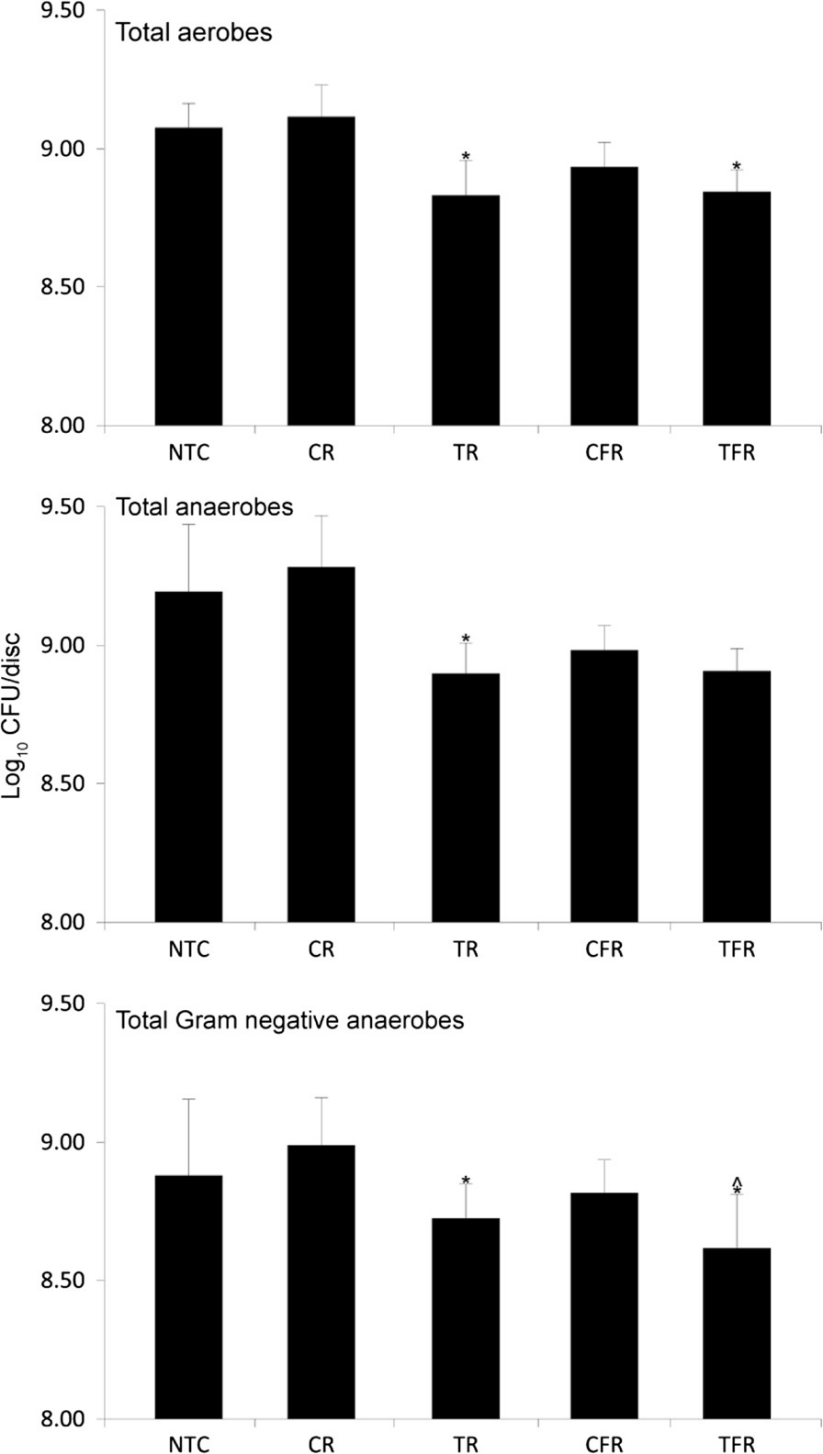
Viability of oral biofilms following repeated exposures to test rinses. Biofilms were cultivated on hydroxyapatite surfaces inoculated with saliva. Biofilms were then exposed, in triplicate, to mouthrinse formulations twice daily for 4 d. Data show mean viability of aerobes/facultative anaerobes total anaerobes and Gram-negative anaerobes as determined by viable counting. Both test rinses caused significant reductions in viability compared to their respective controls (*, *p* < 0.05, *n* = 3), except TFR against anaerobes. CFR caused significant reductions in viability of aerobes compared to treatment-free control (#, *p* < 0.05, *n* = 3). There were no significant differences between TR and TFR except against Gram-negative anaerobes in three separate experiments (^, *p* < 0.05, *n* = 3)

The effects of test rinses on the viability of biofilms formed by salivary bacteria were also compared by dosing biofilms twice daily for four days (Fig. 4). The viability of aerobes/facultative anaerobes, total anaerobes and Gram-negative anaerobes was assessed by viable counting. TR caused significant viability reductions in all groups compared to CR (*p* < 0.05). TFR also caused significant reductions compared to its respective control, CFR, except against total anaerobes. There was no significant difference between the effects of CR and the treatment-free controls. CFR, however, did cause viability reductions and in the case of aerobes, CFR-treated plaques were significantly less viable than a treatment-free control. There was no significant difference between TR and TFR except against Gram-negative anaerobes, where TFR was marginally more effective.

### Pre-treatment of hydroxyapatite inhibits biofilm growth

**Fig. 5:**
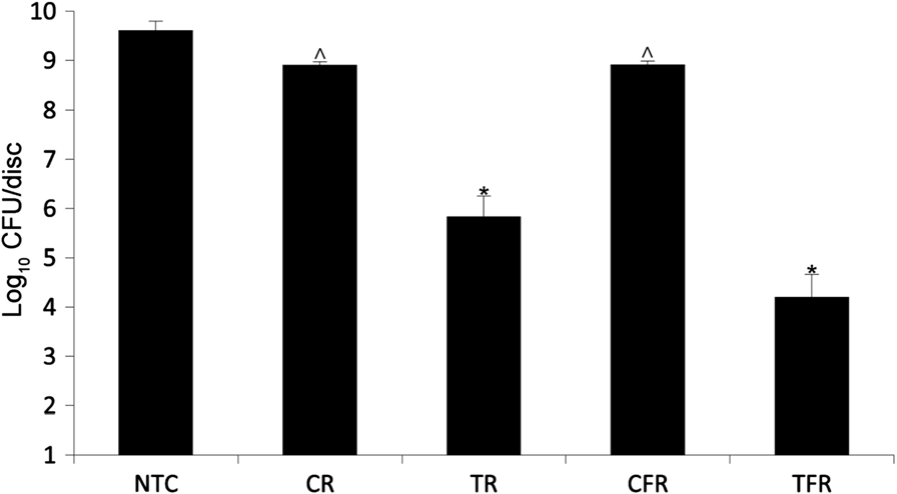
Biofilm growth on hydroxyapatite surfaces pre-exposed to test rinses. Hydroxyapatite discs were soaked in mouthrinse formulations (final concentration, 100 %) containing CPC (TR) or CPC and fluoride (TFR). Control rinses without CPC (CR and CFR, respectively) and a treatment-free control (NTC) were also included. Six replicate discs for each treatment group were included. Biofilms were then cultivated on pre-exposed discs and mean total anaerobes were enumerated by viable counting. Surfaces exposed to both test rinses supported significantly less biofilm growth than their respective negative controls in six separate experiments (*, *p* < 0.05, *n* = 6). Pre-exposure to control rinses also resulted in less dense biofilms, although this effect was modest (^, *p* < 0.05, *n* = 6)

Hydroxyapatite surfaces were pre-exposed to test rinses to assess the effect on subsequent biofilm growth (Fig. 5). Treatment with TR and TFR resulted in large differences in biofilm density compared to their respective controls (3.1 and 4.7logs, respectively, *p* < 0.01). There was no significant difference between the fluoridated and non-fluoridated rinses.

## Discussion

The use of mouthrinses reportedly augments oral hygiene regimens based on regular brushing [12] and is believed to offer enhanced protection against dental caries, particularly when recommended oral healthcare regimens are not strictly adhered to [26]. The incorporation of sodium fluoride into antibacterial mouthwash formulations may further enhance caries control, especially post-brushing, when rinsing with water or a non-fluoridated mouthrinse can remove potentially beneficial residual fluoride [33]. *In vitro* assessments of the activity of mouthwash formulations are instructive, provided that a comprehensive assessment is made. The current investigation therefore employed six distinct methods to assess the antibacterial efficacy of two mouthrinse formulations, one containing 0.075 % CPC (TR) and another with 0.075 % CPC and 250 ppm sodium fluoride (TFR). The methods varied with respect to the test bacteria used, exposure times, the concentrations of the formulations and the types of outcome that were evaluated. Biofilms were cultured under anaerobic conditions in order to replicate the anoxic conditions extant in mature plaques [39].

In terms of mouthrinse effects on bacteria in planktonic culture, direct exposure of populations in this mode of growth indicated that large bactericidal effects occurred following short-term exposures to undiluted mouthrinses reflecting oral concentrations during rinsing *in vivo,* and provides a useful indication of the ability of the formulations to rapidly inactivate bacteria. Two test populations were used; a pure culture of the cariogenic bacterium *S. mutans* and a mixed culture of oral bacteria derived from saliva. Whilst both *S. mutans* cultures and mixed populations of oral bacteria were significantly inactivated by both test formulations, the inclusion of mixed populations is of value because growth in multi-species communities, even without biofilm formation, may decrease bacterial susceptibility. The resazurin dye-based method was used to assess measured metabolic effects of lower concentrations of test formulations on dense populations of mixed oral bacteria or pure cultures of *Actinomyces viscosus* within 10 min. of exposure. These concentrations reflect residual levels of mouthrinse that might be expected to remain in the mouth following rinsing. Furthermore, this rapid method is relatively simple to perform and lends itself to high-throughput analysis, particularly since results correlate well with other approaches. The *ex vivo* test also assessed exposures to a lower concentration of test rinse, but this approach used fresh samples of oral bacteria isolated directly from human volunteers. These data showed good reproducibility between five volunteers, indicating that such techniques may provide useful preliminary data for larger clinical studies.

Antibacterial effects on biofilms were determined using three methods: i) Profiling by confocal microscopy [40, 41], which enabled the visualisation of cellular viability through plaque biofilms following a single exposure. This technique facilitates analysis of the spatial distribution of viable biomass within intact plaque. The creation of intensity plots in the z axis, although not necessarily representative of the thickness of the overall biofilm, indicates the extent of penetration of the antimicrobial compound through the sample. It is notable that, even in the depths of larger biofilms clusters, considerable bacterial inactivation is apparent. This highlights the utility of confocal depth profiling versus epifluorescence microscopy, which provides a top-down view of overall biofilms viability. ii) Viable counting of treated plaques was also utilised and determines changes in the viability of different functional groups of bacteria in similarly-cultured plaques. Such changes can be quantified following multiple exposures, revealing longer-term effects of test formulations. iii) The activity of test formulations was also assessed by pre-treating hydroxyapatite surfaces prior to inoculation. Data thus generated indicate whether components of test formulations adsorbed onto surfaces are able to inhibit cell attachment or biofilm maturation, with obvious relevance to oral hygiene.

CPC-containing test rinses with (TFR) and without (TR) fluoride consistently and significantly inactivated bacteria in planktonic, *ex-vivo* and biofilm models. TR and TFR were both highly bactericidal against cultures of the cariogenic bacteria *S. mutans* and *A. viscosus*, against salivary oral bacteria, and against oral rinse samples from human volunteers. Both mouthrinses caused marked and significant viability losses even at concentrations of 5 %, suggesting that residual levels of test rinse may exhibit continued efficacy after rinsing.

Single and multiple exposures of plaque biofilms to both test rinses caused marked viability changes; confocal microscopy revealed extensive viability reductions in three dimensions and viable counting showed reductions in all bacterial groups tested. Both test rinses displayed a significant biofilm-inhibitory effect when used to pre-treat hydroxyapatite surfaces although it remains to be determined whether attachment, maturation or cell growth rate was inhibited.

With respect to the effect of fluoride on the efficacy of CPC-containing rinses, TFR and TR generally exhibited equivalent activities. This indicates that the presence of fluoride at 225 ppm does not reduce the antibacterial efficacy of mouthrinse formulations containing CPC. Combined with previous findings that incorporation of CPC does not affect the ability of fluoride to inhibit demineralisation, these data support the combined use of CPC and fluoride in mouthrinse formulations.

## Conclusions

CPC-containing mouthrinses, with and without fluoride, exhibited significant antibacterial efficacy against oral bacteria in planktonic and biofilm modes, and at varying concentrations. Fluoridated and non-fluoridated CPC rinses generally exhibited equivalent activities, supporting the combined use of CPC and sodium fluoride in mouthrinse formulations.

## Methods

### Saliva samples

In all experiments where saliva was used as inoculum to generate planktonic cultures of oral bacteria or *in vitro* dental plaques; or where saliva was analyzed following *ex vivo* exposure, the volunteers (2 M, 3 F without active cares or periodontal disease), were instructed to brush as normal twice a day with a standard fluoride toothpaste without additional oral hygiene for seven days prior to saliva collection. Without exception, saliva was used within 1 h of collection.

### Test formulations

Two mouthrinse formulations containing 0.075 % cetylpyridinium chloride (CPC) with or without sodium fluoride (225 ppm) (TFR and TR, respectively) were tested in this study. Otherwise-identical controls without CPC were also included (with or without sodium fluoride, CFR and CR, respectively).

### Direct planktonic exposures

Cultures (20 ml) of *S. mutans* or salivary bacteria (fresh human saliva diluted 1:100 in Wilkins Chalgren broth and incubated immediately after collection), were grown statically in Wilkins Chalgren broth at 37 °C to OD_600_ 0.8 aerobically. Aliquots (1 ml) were centrifuged, the pellet resuspended in test formulations (100 %, 1 ml) and incubated at room temperature with shaking for 30 s. Suspensions were centrifuged and washed twice in phosphate buffered saline (PBS; 0.01 M phosphate, 0.0027 M KCl, 0.137 M NaCl, pH 7.4). Serial dilutions were performed in PBS, spread-plated on Wilkins Chalgren agar and incubated aerobically at 37 °C for viable counts.

### Immediate effects on bacterial metabolism

The effects of test rinses on the redox activity of oral bacteria was assessed using a resazurin-based dye, in an adaptation of a method initially described by Shiloh and co-workers [42]. Cultures (*n* = 3) of *A. viscosus* or salivary bacteria (saliva diluted 1:100 and cultured aerobically at 37 °C to OD_600_ 0.8 in tryptone soy broth, which was shown to be the most suitable medium in validation experiments (data not shown)) were used. These were exposed to test mouthrinses for 10s (final concentration, 10 %) before immediately adding alamarBlue™ viability indicator (Life Technologies, Paisley, U.K.) to a final concentration of 6 % v/v). Following incubation at room temperature for 10 min, cultures were examined visually and spectrophotometrically (mean background-corrected A_570_-A_600_ values) to assess viability.

### *Ex vivo* tests

Healthy volunteers (*n* = 5, 2 males and 3 females, age range 24–35) were asked to refrain from oral hygiene for 12 h before sampling. *Ex vivo* samples were then acquired by asking volunteers to rinse their mouths with 10 ml of natural mineral water (Sainsbury’s, London, UK), expectorate into a sterile test tube, brush their tongues with a new soft brush and agitate in the water, to generate suspensions containing oral bacteria. Mouthrinse formulations (500 μl) were added to each suspension (10 ml, final concentration, 5 % v/v) and incubated for 1 min before performing serial dilutions and spread-plating on Wilkins Chalgren agar for viable counts.

### Confocal microscopy of dosed biofilms

Optical sectioning of viability distributions in plaque was carried out using techniques developed by Netuschil *et al.* [43] and Hope and co-workers [40] and adapted by Ledder *et al.* [41]. In the current study, plaque biofilms were grown and maintained in a hydroxyapatite disc biofilm reactor as previously described [44] and adapted as follows: Wilkins Chalgren broth containing 0.25 % porcine mucin and 1 % sucrose (0.5 ml) was dispensed into a sterile 24-well tissue culture plate. The broth was then inoculated with saliva (0.5 ml). Sterile hydroxyapatite discs were placed in the wells and incubated anaerobically for 24 h at 37 °C. Biofilms were removed from the anaerobic workstation and exposed once at room temperature to test mouthrinses or sterile water (no-treatment control) for 2 min. All plaques were gently rinsed with sterile water to remove excess treatments, stained with a combination of fluorescent dyes i.e. propidium iodide and SYTO9 (Live/Dead stain) and evaluated by confocal scanning laser microscopy (TCS SP5, Leica Microsystems, Milton Keynes, UK) for microbial viability profiling. The entire disc was evaluated visually and representative images (1 per disc) were captured with LAS AF software (Leica), covering the entire depth of the biofilm. Further processing, quantification and depth profiling was performed using Imaris (Bitplane AG, Zurich, Switzerland).

### Plaque viability tests

Plaque biofilms were maintained in a hydroxyapatite disc biofilm reactor as described above prior to the following dosing regimen: Twice daily for 2 min., discs were transferred to fresh plates containing test rinse or sterile water (2 ml). In order to maximise microbial diversity, the discs were then transferred to a fresh plate containing broth (morning) or broth and fresh salivary inoculum (evening). This dosing regimen continued for 4 days in order to broadly simulate the repeated exposure and regrowth cycle which would be experienced by plaque *in-vivo*. Following the final dosing, discs were aseptically removed, gently immersed in sterile PBS to remove loosely attached organisms, added to pre-reduced, half-strength thioglycollate medium and vortexed thoroughly for 1 min. Samples were serially diluted and appropriate dilutions (0.1 ml) were plated in triplicate onto a variety of selective and non-selective media. These media were as follows; Wilkins-Chalgren agar (incubated anaerobically for total anaerobes or aerobically for total aerobes and facultative anaerobes) and Wilkins-Chalgren agar with gram-negative supplement (total gram-negative anaerobes). Inoculated agars were incubated in an anaerobic chamber (MG1000 workstation, Don Whitley Scientific, UK) at 37 °C for up to 5d. Aerobes were incubated aerobically for up to 3d. Three biological replicates were included, each analysed in triplicate.

### Inhibition of bacterial attachment to hydroxyapatite

Hydroxyapatite discs (*n* = 6) were incubated statically at room temperature in test rinses or sterile water (1 ml in the wells of a 12-well tissue culture plate) for 24 h. Discs were then rinsed briefly in distilled water and transferred to a sterile 24-well tissue culture plate with Wilkins Chalgren broth containing 0.25 % porcine mucin and 1 % sucrose (0.5 ml). Medium was then inoculated with saliva (0.5 ml). Plaques were incubated anaerobically at 37 °C for 48 h, changing the medium at 24 h. Following this growth phase, discs were rinsed and suspended in half-strength thioglycollate medium, before performing serial dilutions and spread-plating on Wilkins Chalgren agar for viable counts of total anaerobes as described above.

### Ethics and consent

Advice was taken from the Chair of a University of Manchester Research Ethics Committee regarding the correct procedures associated with the use of human oral rinse samples for the *ex vivo* experiments. The committee granted exemption from formal ethics approval due to the nature of the work but as advised, informed consent was obtained from all volunteers and all samples were collected anonymously.

### Data analysis

All data are shown as mean values plus/minus standard deviations. Statistical significance between data sets was calculated using Student’s *t*-test with significance reported at ≤0.05 (Microsoft Excel 2010).

## Abbreviations

CPC: Cetylpyridinium chloride
TR: Test rinse
TFR: Test fluoride rinse
CR: Control rinse
CFR: Control fluoride rinse
NTC: Treatment free control
